# Water stress and insect herbivory interactively reduce crop yield while the insect pollination benefit is conserved

**DOI:** 10.1111/gcb.15386

**Published:** 2020-10-29

**Authors:** Chloé A. Raderschall, Giulia Vico, Ola Lundin, Astrid R. Taylor, Riccardo Bommarco

**Affiliations:** ^1^ Department of Ecology Swedish University of Agricultural Sciences Uppsala Sweden; ^2^ Department of Crop Production Ecology Swedish University of Agricultural Sciences Uppsala Sweden

**Keywords:** climate change, drought, faba bean, herbivory, insect pollination, water stress

## Abstract

Climate change is predicted to hamper crop production due to precipitation deficits and warmer temperatures inducing both water stress and increasing herbivory due to more abundant insect pests. Consequently, crop yields will be impacted simultaneously by abiotic and biotic stressors. Extensive yield losses due to such climate change stressors might, however, be mitigated by ecosystem services such as insect pollination. We examined the single and combined effects of water stress, insect herbivory and insect pollination on faba bean yield components and above‐ and belowground plant biomass under realistic field conditions. We used rainout shelters to simulate a scenario in line with climate change projections, with adequate water supply at sowing followed by a long period without precipitation. This induced a gradually increasing water stress, culminating around crop flowering and yield formation. We found that gradually increasing water stress combined with insect herbivory by aphids interactively shaped yield in faba beans. Individually, aphid herbivory reduced yield by 79% and water stress reduced yield by 52%. However, the combined effect of water stress and aphid herbivory reduced yield less (84%) than the sum of the individual stressor effects. In contrast, insect pollination increased yield by 68% independently of water availability and insect herbivory. Our results suggest that yield losses can be greatly reduced when both water stress and insect herbivory are reduced simultaneously. In contrast, reducing only one stressor has negligible benefits on yield as long as the crop is suffering from the other stressor. We call for further exploration of interactions among ecosystem services and biotic and abiotic stressors that simulate realistic conditions under climate change.

## Graphical abstract

Climate change is predicted to hamper crop production by inducing a range of abiotic and biotic stressors that interact with ecosystem services. We examined the single and combined effects of water stress, insect herbivory and insect pollination on faba bean yield components. Our results suggest that yield losses can be greatly reduced when both water stress and insect herbivory are reduced simultaneously. Insect pollination had a constant positive effect on yield, highlighting the importance of managing agricultural landscapes to support pollinators irrespective of water stress and insect herbivory.

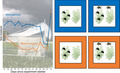

## 1 INTRODUCTION

Agricultural crop production is expected to be increasingly affected by weather patterns associated with climate change, such as more frequent prolonged dry periods (Lesk et al., 2016; Rosenzweig et al., 2001; Seneviratne et al., 2012). Water stress resulting from insufficient precipitation during the growing season causes major yield losses in most cultivated crops (Daryanto et al., 2015; Leng & Hall, 2019; Olesen et al., 2011). In addition, crop yields in temperate regions are predicted to be increasingly impacted by insect herbivory as a warming climate promotes insect pest population growth, consumption rates (Deutsch et al., 2018; Lehmann et al., 2020) and range expansion into cooler, hitherto unsuitable regions (Bebber et al., 2013). According to the plant stress hypothesis, insect herbivore populations are expected to increase on water‐stressed plants due to increased resource (e.g. nitrogen) availability (White, 1969, 1974). However, empirical outcomes are variable with insect herbivory both increasing and decreasing on water‐stressed plants (Huberty & Denno, 2004; Larsson, 1989; Price, 1991; Tariq et al., 2012). It thus remains inconclusive how water stress and insect herbivory simultaneously affect crop yield, because the effect of water stress on insect herbivore performance likely determines if yield loss in response to insect herbivory is less or more pronounced in water‐stressed plants (Fereres et al., 1988; Oswald & Brewer, 1997; Simpson et al., 2012).

While abiotic and biotic stressors reduce crop yields, crop yields can also benefit from a suite of mutualistic biotic interactions. Insect pollination, for example, significantly enhances yield of approximately 75% of the globally important crop species (e.g. Klein et al., 2007). There is, however, little empirical evidence of how the insect pollination benefit on yield components is affected by biotic and abiotic stressors (but see Bishop et al., 2016, 2017, for the effects of heat stress on pollination). Insect pollination and insect herbivory often interactively shape crop yields and, more specifically, pollination benefits are frequently enhanced when insect herbivores are effectively suppressed (Garibaldi et al., 2018; Tamburini et al., 2019). Simultaneous effects of water availability and insect pollination on yield have mostly been investigated from an irrigation management perspective, with water availability levels representing conventional and reduced irrigation regimes (Fijen et al., 2020; Hebblethwaite et al., 1984; Klein et al., 2015; Stoddard, 1986). Under different irrigation levels, water availability and insect pollination affect yield both synergistically (Klein et al., 2015) and additively (Fijen et al., 2020; Hebblethwaite et al., 1984). However, the irrigation regimes explored represent different levels of recurring water inputs rather than simulating a dry spell caused by infrequent rainfall events, as is increasingly the reality for rainfed agriculture (Seneviratne et al., 2012). Since 80% of the global cropland is rainfed agriculture (Ramankutty et al., 2018) and irrigated agriculture is not always a feasible or sustainable approach, we need to quantify the level of plant water stress induced by precipitation patterns similar to those expected under climate change; and how such water stress in combination with biotic stressors and mutualists affect crop yield. To date, interactive effects between water stress and insect pollination on crop yield have only been examined once, and no interactions were found (Groeneveld et al., 2010). Yet, flowers were pollinated by hand (Groeneveld et al., 2010), which does not consider potential changes in the interaction between plants and their insect pollinators in response to stressors.

Water stress and insect herbivory can interfere with the mutualistic relationship between plants and pollinators by altering the quality or quantity of floral rewards. Floral rewards determine flower attractiveness and influence pollinator visitation rate and pollination (Potts et al., 2003). Plants that suffer from water stress (Caruso, 2006; Waser & Price, 2016) or insect herbivory (Jacobsen & Raguso, 2018; Mothershead & Marquis, 2000; Strauss et al., 2002) often produce fewer flowers, as well as reduced pollen and nectar quantity and quality. Reduced pollinator visitation rate due to stress‐induced alteration of floral rewards can lead to lower seed‐set (Gallagher & Campbell, 2017). But yield is not only limited by reduced pollinator visitation rates: changes in pollen and nectar quantity and quality can also influence pollinator foraging behaviour (Adler, 2000). Bees can behave differently when foraging for pollen and nectar (Sprengel, 1793); they perform legitimate pollination visits, whereby bees insert the proboscis into the flower tube to collect pollen and nectar, thereby transferring pollen to the stigma (Tasei, 1976). In addition, some bees rob nectar by extracting it from a hole they bite in the flower tube and without getting in contact with the flowers' sexual parts (Inouye, 1980; Tasei, 1976). Switches in bee foraging behaviour are associated with pollen and nectar availability, where legitimate pollination increases when pollen abundance and quality are high (Marzinzig et al., 2018; Poulsen, 1973). It remains unknown if water stress or insect herbivory trigger behavioural shifts that indirectly affect crop yield by altering foraging behaviour.

We examined the single and combined effects of water stress, insect herbivory by aphids and insect pollination by bumble bees on faba bean yield components and above‐ and belowground plant biomass under realistic field conditions. We used rainout shelters to simulate a scenario with adequate water supply at sowing, followed by a long period without precipitation, in line with climate change projections. This gradually increasing water stress over the growing season culminated around flowering and early pod‐fill, when the crop is most sensitive to reduced water availability (Daryanto et al., 2015; Karkanis et al., 2018). To understand the mechanisms underlying treatment effects on faba bean plants and yield components, we monitored soil water availability, aphid abundances and bumble bee flower visitation rate and foraging behaviour. We hypothesized that the simultaneous impacts of abiotic (water stress) and biotic (insect herbivory) stressors interact to reduce crop yield more than their individual impacts due to improved herbivore performance on water‐stressed plants. Further, we hypothesized that the insect pollination benefit to yield (kg/ha) is less pronounced in plants suffering from water stress and insect herbivory than in healthy plants, due to reduced pollinator visitation and a behavioural shift from legitimate flower visits to nectar robbing.

## 2 MATERIALS AND METHODS

### 2.1 Study system

Faba bean (*Vicia faba* minor L.) is one of the most important legume crops worldwide but its global acreage has been declining since 1980 due to its yield instability (Karkanis et al., 2018). Faba bean is sensitive to water stress (Ghassemi‐Golezani et al., 2009; Katerji et al., 2011; Khan et al., 2007). Biotic factors influencing faba bean yield include its partial dependence on insect pollination (Cunningham & Le Feuvre, 2013; Free, 1966; Suso et al., 1996) and its susceptibility to a range of insect pests of which aphids are considered the most damaging (Hansen et al., 2008; Stoddard et al., 2010). In particular, the black bean aphid (*Aphis fabae* Scopoli) can cause severe yield losses by feeding on the phloem and by serving as vectors of a variety of plant diseases (Hansen et al., 2008; Stoddard et al., 2010).

### 2.2 Experimental design

**FIGURE 1:**
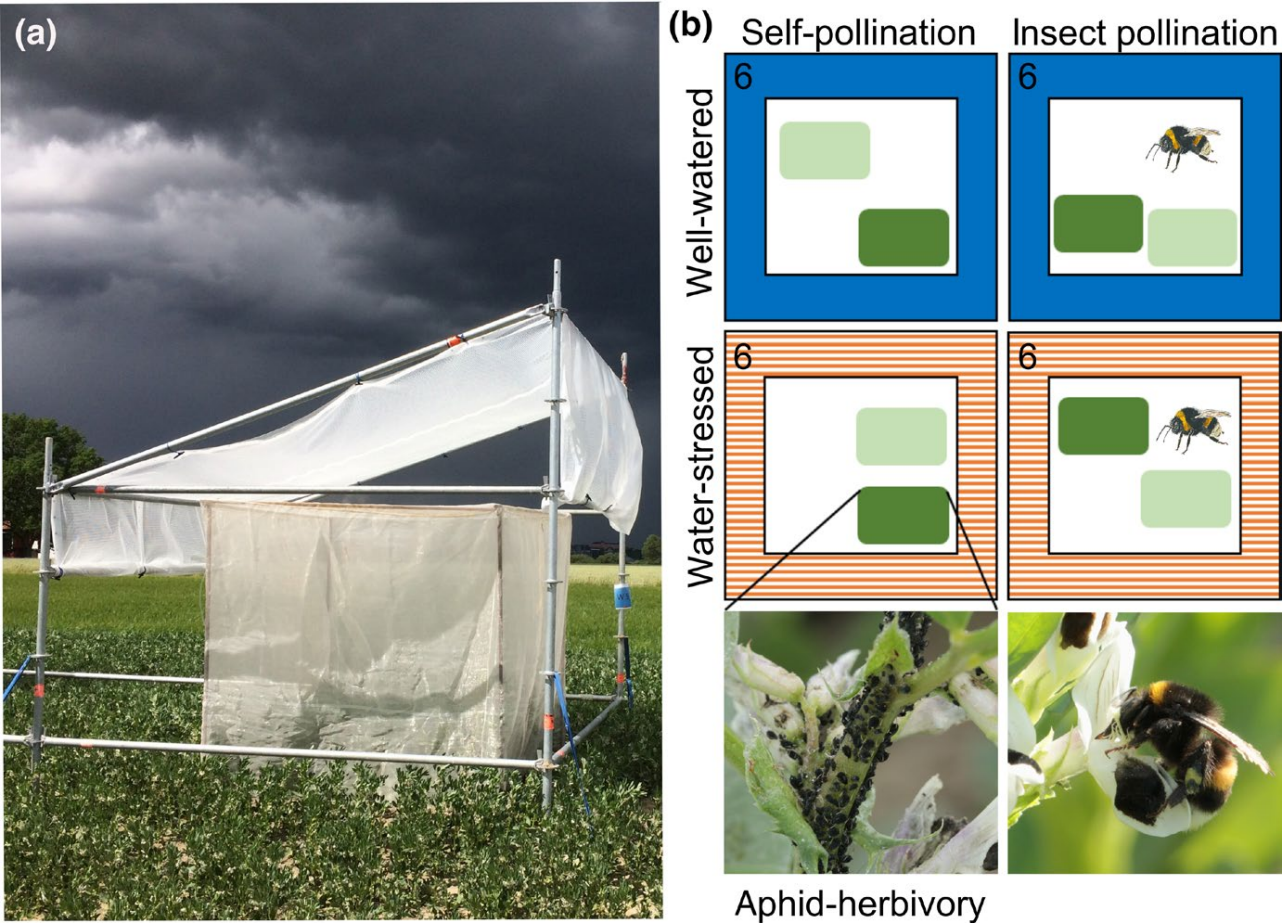
Experimental design showing a rainout shelter with a cage underneath (a) and a schematic of the different treatments contained in each of the six replicated blocks (b): well‐watered plots (blue, solid), water‐stressed plots (orange, striped), insect‐pollinated plots (white with bumble bee) and self‐pollinated plots (white without bumble bee). Nested within each block, we set up a split‐plot design containing one subplot with 10 aphid‐infested plants (dark green), and one subplot with 10 aphid‐free plants (light green). The number 6 indicates the number of replications for each water availability and pollination treatment combination. Photos of a single plant infested with black bean aphids (*Aphis fabae*), and of a faba bean flower being legitimately pollinated by a buff‐tailed bumble bee worker (*Bombus terrestris*)

We performed a field experiment in a faba bean field near Uppsala, Sweden (latitude: 59.83°N, longitude: 17.70°E), in 2019. Climatic conditions during spring and summer 2019 were similar to the long‐term average conditions in the region (Table S1). The soil type at the site was a light clay, with clay content ranging between 10% and 19% depending on the exact location in the field. In early May, an area of 0.1 ha was sown with the commonly cropped faba bean cultivar Tiffany (Svenska Foder) using 55 seeds per m^2^. Upon plant emergence (BBCH 14; Weber & Bleiholder, 1990), we erected 24 cages, each 2 by 2 by 2 m covered with a net (Artes Politecnica SRL), with a mesh size of 0.35 by 1.6 mm; the net was dug approximately 10 cm into the soil to prevent insects from leaving or entering (Figure 1a). The cages were placed under rainout shelters, and water supply was manipulated to obtain two water availability treatments (well‐watered vs. increasingly water‐stressed; see below). We used a randomized complete block design replicated in six blocks for the water availability treatment crossed with the pollination treatment (insect pollination vs. self‐pollination; Figure 1b). Nested within the block design, we set up a split‐plot design for the insect herbivory treatment, in which each cage contained two subplots assigned to one of two insect herbivory treatments, that is, 24 aphid‐free and 24 aphid‐infested subplots (Figure 1b). Experimental plots were treated with 0.5 kg/ha of the fungicide Signum (BASF; 267 g/kg boscalid + 67 g/kg pyraclostrobin) shortly before bloom (BBCH 59) on 18 June.

### 2.3 Water availability treatment

To simulate a long dry spell, in line with projections of more intermittent precipitation events due to climate change (Seneviratne et al., 2012), we excluded natural precipitation between 31 May, when most of the plants across experimental plots had four leaf pairs unfolded (BBCH 14), and 17 July, when plants reached mid pod fill stage (BBCH 73), that is, for 46 days. Over the past 70 growing seasons (May–August) in the region, dry spells, defined as number of consecutive days with precipitation below 1 mm, had a mean duration of 11.5 days, and ranged between 4 days in 1950 and 37 days in 2018. Hence, the duration of the experimental precipitation exclusion is longer than what would be expected under the current typical local climatic conditions. It is, however, relevant in the face of climate change, where the extremely dry summer of 2018 in central and northern Europe is expected to become the norm by mid‐century (Toreti et al., 2019). The period of precipitation exclusion was chosen to induce the largest water stress during the faba bean flowering to pod‐fill stage, which is the crop's most sensitive growth stage for water stress (Daryanto et al., 2015; Karkanis et al., 2018).

Natural precipitation was completely excluded using rainout shelters. The shelter roofs consisted of transparent reinforced plastic material (Layher) with a density of 200 g/m^2^ and light transmission of 70%, fixed at an angle of approximately 20°. The roofs were fixed on top of a 3 by 3 m scaffold construction, which was 3 m high in the front and 2 m high in the back (Figure 1a). We oriented shelters with the lower roof side towards the prevailing wind direction during summer (210°) to minimize border effects from rain falling on the sides of experimental plots. The same constructions were built for both the well‐watered and the water‐stressed treatments. The water‐stressed treatment did not receive any water while the rainout shelters were in place. Conversely, the well‐watered treatment was regularly irrigated such that water losses due to evapotranspiration were replaced. To keep soil moisture levels nearly stable, each plot was watered approximately with the amount the plants lost through evapotranspiration during the previous week, which was 22.5 mm, as estimated using FAO CROPWAT 8.0, which uses the Penman–Monteith formula (Allen et al., 1998). In order to minimize run‐off, we watered cages three times per week with 7.5 mm/m^2^. In the end, the total monthly water inputs were in line with precipitation averages in the region (Table S1). To limit fungal diseases or disturbance of the aphid colonies, we watered plots in between faba bean plants and below the leaf canopy with watering cans.

To quantify the effects of the two water availability treatments on plant available water, soil volumetric water content was monitored twice per week in one well‐watered and one water‐stressed plot in each block using a PR2/4 profile probe (Delta‐T Devices Ltd.). Measurements were taken at the centre of the experimental plot at 10, 20, 30 and 40 cm soil depth; and provided soil volumetric water content data extending approximately 6–10 cm horizontally into the soil (Figure S1). In addition, to estimate the soil moisture corresponding to the plant wilting point, we reconstructed the site‐specific soil water retention curve (Figure S2) by simultaneously measuring soil water potential and soil volumetric water content in one location outside the rainout shelters. For this, we used an MPS‐6 dielectric water potential sensor and a GS3 soil water content and soil temperature sensor (Decagon Devices, Inc.), which we co‐located at 10 cm depth during the period 7 July–5 August. Plants were also visually checked regularly and did not exhibit any signs of wilting even at the lowest soil water levels.

### 2.4 Insect herbivory treatment

At the onset of faba bean flowering, we inoculated a randomly chosen cluster of 10 plants in each cage with 10 similarly sized wingless adult female black bean aphids (*Aphis fabae*) that had been reared on faba bean plants in the greenhouse. However, many aphids were immediately eaten by arthropod predators that remained in the cages. In order to obtain similar aphid‐infestation on all 10 plants per cage, we removed any arthropod predators inside the cages and re‐inoculated plants on which aphids had not established over the following days until aphid colonies started to grow on all plants. The black bean aphids used for the re‐inoculation were collected from a nearby faba bean field and varied in size, but the size variation was comparable across all re‐inoculated plants. Aphid abundances were monitored every week on the 10 aphid‐infested and 10 aphid‐free plants by assigning one infestation class to each plant: 0: 0 aphids, 1: 1–5, 2: 6–25, 3: 26–125, 4: 126–625 and 5: >625 aphids (Grechi et al., 2008). Any aphids that established on control (aphid‐free) plants were removed. In some cases, where large colonies of aphids had infested control plants, we re‐assigned a new randomly selected plant that was free of aphids as control. In total, we monitored aphid abundances on 11 occasions between 27 June and 13 August.

### 2.5 Pollination treatment

Shortly before the onset of faba bean flowering (BBCH 59), half the cages were supplemented with one hive of buff‐tailed bumble bees (*Bombus terrestris*) consisting of approximately 10 workers and male brood (Natupol Seeds; Koppert Biological Systems). *B. terrestris* is a potentially less efficient pollinator of faba bean compared with long‐tongued bumble bee species because besides legitimately pollinating flowers, it can also rob nectar (Marzinzig et al., 2018; Tasei, 1976). *B. terrestris* is, however, one of the most common pollinators of faba bean in Scandinavia (Poulsen, 1973) and is the only bumble bee species that is commercially available. Bumble bees were only allowed to forage within the cages. To avoid that bumble bees visited unrealistically many faba bean flowers, or had insufficient food available, we supplemented each colony with sugar water and sterilized pollen. The sugar water was available inside the hive, whereas the pollen was supplied outside the hive to motivate bumble bees to forage within the cage. Given the experimental design, bumble bees could either visit aphid‐infested or aphid‐free plants or feed on the pollen and sugar water we provided, but they were not able to choose between well‐watered or water‐stressed plants since all plants within a cage were subject to the same water availability treatment.

### 2.6 Pollinator visitation rate

In the cages with bumble bee hives, we assessed pollinator flower visitation rate eight times during faba bean flowering between 29 June and 10 July. Pollinators were monitored between 11:00 and 17:00 hr on days with temperatures above 15°C. We counted the number of flower visitations to the 10 aphid‐infested and the 10 aphid‐free plants for 10 min. For each visit, we noted plant identity and whether pollinators were legitimately pollinating flowers by inserting their proboscis through the front of the flower opening, or robbing nectar by inserting their proboscis through a hole at the base of the flower tube to extract nectar. Pollinator visits to extra‐floral nectaries, which are located on stipules below flowers, were not considered since they do not contribute to pollination. At the end of each observation session, we counted all open flowers on the 10 aphid‐infested and the 10 aphid‐free plants.

### 2.7 Yield components, above‐ and belowground plant biomass

Prior to harvesting plants, we estimated plant density by counting the number of plants within a 0.36 m^2^ quadrat randomly placed in each cage. When pods reached maturity, we manually harvested plants from both insect herbivory treatments in each cage (10 aphid‐infested and 10 aphid‐free plants). We also excavated the root system of all 20 plants in each cage. Maximum vertical length of the tap root was measured for each plant immediately upon excavation. In the laboratory, we separated the root system from the aboveground plant biomass. Aboveground plant biomass, pods and beans were oven‐dried at 65℃ for 48 hr. Subsequently, pods per plant and beans per pod were counted and aboveground plant biomass (including leaves, stems and pod husks) and beans were weighed separately. Roots were gently washed with water and subsequently dried at 65℃ for 48 hr and then weighed. Yield was calculated by multiplying average bean mass per plant in each cage with crop plant density per quadrat, and then recalculated and expressed as kg dry beans per hectare. We assumed equal crop plant density for aphid‐infested and aphid‐free plants. The harvest index was calculated by dividing bean mass by aboveground plant biomass plus bean mass.

### 2.8 Statistical analysis

All statistical analyses were done using linear mixed‐effect models (package ‘lme4’; Bates et al., 2015) in R version 3.6.1 for Windows (R Core Team, 2019). The amount of variance that contributed to a sample by different factors was analysed with a type III ANOVA (package ‘lmerTest’; Kuznetsova et al., 2017) and the denominator degrees of freedom were calculated using the Kenward–Roger's method (package ‘pbkrts’; Halekoh & Højsgaard, 2014). We visually examined the residuals of each model to check that model assumptions were met.

To analyse faba bean yield and biomass parameters, we used bean yield per unit area, bean mass per plant, number of pods per plant, number of beans per pod, individual bean weight, harvest index, aboveground plant biomass, root length and root biomass as response variables. To avoid transformation of response variables or using generalized models with link functions, which both might qualitatively affect ordinal interactions (Berrington de González & Cox, 2007), we averaged data of all response variables to subplot level (aphid herbivory treatment within each cage) and used normal distributions without transformations for all response variables. The only exception was foraging behaviour, which was transformed to meet model assumptions, but we verified that the qualitative results for this variable were conserved when analysed on the linear scale (untransformed). We included the three treatments water availability, insect herbivory and pollination and their two‐ and three‐way interactions, as fixed effects in the models. No model simplification was done, because treatment interactions were an inherent part of the study design. Cage identity nested within block identity was added as a random effect.

Aphid densities were analysed by calculating the mean aphid infestation category, averaged over the 11 sampling occasions and 10 aphid infested plants in each cage as response variable. As fixed effects, we used water availability and pollination treatments and their interaction. We used normal distributions without transformations. Block identity was added as the random effect.

Pollinator visitation rate was analysed for all cages containing bumble bee hives. As response variables, we used flower abundance, bumble bee visitation rate per flower for each foraging behaviour (legitimate pollination and nectar robbing) averaged to subplot‐level (aphid herbivory treatment within each cage). We used a normal distribution without transformations for flower abundance per plant. Foraging behaviours per flower were log‐transformed to meet model assumptions. As fixed effects, we used water availability and insect herbivory treatments and their interaction. Cage identity nested within block identity was added as the random effect.

## 3 RESULTS

### 3.1 Soil water availability

We experimentally subjected faba beans to 46 days without precipitation. As a result, soil volumetric water content continuously declined in the water stress treatment from 15% to 9% and 24% to 17% at 10 and 20 cm soil depth respectively (Figure S1), until it was close to reaching the theoretical permanent wilting point (Figure S2). Yet, we did not see any signs of plant wilting at any point during the experiment. In the well‐watered treatment, we succeeded in keeping soil volumetric water content approximately constant at 18% and 21% at 10 and 20 cm soil depth respectively (Figure S1). After 46 days, the rainout shelters were removed and soil volumetric water content converged between treatments, following a rainfall event that replenished soil water content in both treatments and ensured well‐watered conditions until harvest (Figure S1).

### 3.2 Yield per hectare and bean mass per plant

**FIGURE 2:**
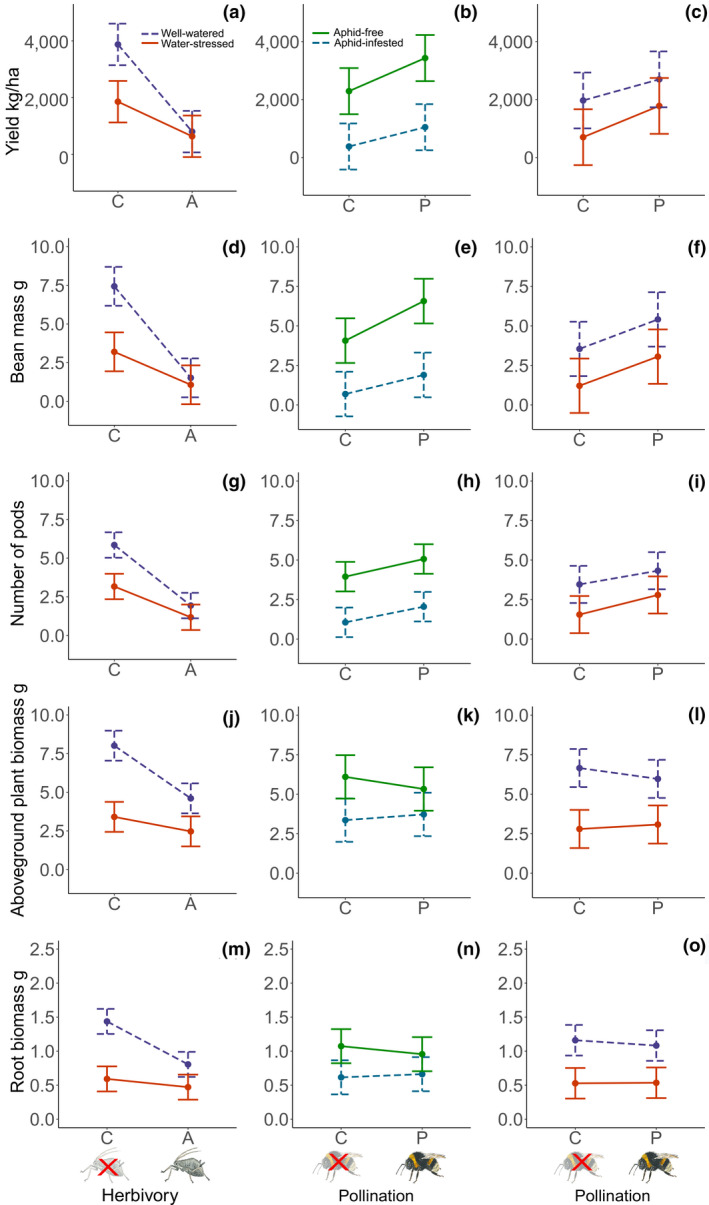
Yield per hectare (a–c), bean mass (grams) per plant (d–f), number of pods per plant (g–i), aboveground plant biomass (grams) per plant (j–l) and root biomass (grams) per plant (m–o) in relation to water availability and herbivory levels (a, d, g, j, m), herbivory and pollination levels (b, e, h, k, n), and water availability and pollination levels (c, f, i, l, o). Well‐watered (blue, dashed), water‐stressed (orange, continuous). Aphid‐free (green, continuous, crossed‐out aphid symbol) and aphid‐infested (turquoise, dashed, aphid symbol). Insect pollination (bumble bee symbol), self‐pollination (crossed‐out bumble bee symbol). Whiskers represent 95% confidence intervals

Yield was explained by an interaction between water availability and insect herbivory. Aphid herbivory reduced yield by 79% in well‐watered plants and water stress reduced yield by 52% in the absence of aphid herbivory (Figure 2a; Table 1). However, the combined effect of aphid herbivory and water stress reduced yield less (84%) than the sum of the individual effects (Figure 2a; Table 1). Insect pollination increased yield by 68% independently of insect herbivory (Figure 2b) and water availability (Figure 2c; Table 1).

**TABLE 1:** Yield components, plant biomass (aboveground), harvest index, root biomass and length, aphid category, flower abundance, legitimate pollination visits and robbing visits per flower with respect to water treatment, pollination treatment and herbivory treatment and their two‐ and three‐way interactions. Shown are *F*‐values (*F*), difference of mean estimates (*e*) for the respective treatments (W = well‐watered, P = insect pollinated, C = aphid‐free), and *p*‐values (*p*)

	Water (W)			Pollination (P)			Herbivory (H)		
	*F*	*e* (W)	*p*	*F*	*e* (P)	*p*	*F*	*e* (C)	*p*
Yield (kg/ha)	11.95	506.34	**.0035**	8.18	1,007.53	**.012**	109.50	1,149.31	**<.001**
Bean mass (g)	21.62	0.94	**<.001**	13.55	1.70	**.0022**	177.01	1.98	**<.001**
Number of pods	25.78	1.35	**<.001**	9.66	1.58	**.0072**	154.43	2.33	**<.001**
Beans per pod	1.63	0.20	.22	10.48	0.45	**.0056**	12.06	0.24	**.0026**
Ind. bean weight (g)	1.94	<0.01	.18	0.79	0.04	.39	58.56	0.14	**<.001**
Plant biomass (g)	52.73	2.44	**<.001**	0.19	0.67	.67	95.03	1.33	**<.001**
Harvest index	0.03	0.07	.86	33.32	0.23	**<.001**	167.41	0.32	**<.001**
Root biomass (g)	56.88	0.37	**<.001**	0.21	0.08	.66	43.34	0.20	**<.001**
Root length (cm)	1.89	1.26	.19	0.65	1.06	.43	2.88	0.28	.11
Aphid category	5.75	0.28	**.030**	0.54	−0.24	.47	‐	‐	‐
Flower abundance	1.39	1.96	.27	‐	‐	‐	19.19	−0.15	**<.001**
log Poll per flower	1.87	−0.75	.23	‐	‐	‐	1.38	0.04	.27
log Rob per flower	0.90	−0.34	.39	‐	‐	‐	8.31	0.73	**.016**

	W*P			W*H			P*H			W*P*H		
	*F*	*e* (W, P)	*p*	*F*	*e* (W, C)	*p*	*F*	*e* (P, C)	*p*	*F*	*e* (W, P, C)	*p*
Yield (kg/ha)	0.31	−682.72	.59	20.41	1522.25	**<.001**	1.34	142.64	.26	0.66	665.36	.43
Bean mass (g)	<0.01	−0.97	.98	39.20	2.79	**<.001**	4.56	0.30	**.045**	2.71	1.99	.12
Number of pods	0.31	−1.19	.59	16.38	1.11	**<.001**	0.07	−0.68	.79	2.90	1.62	.10
Beans per pod	0.62	−0.19	.44	0.26	0.08	.62	0.10	0.05	.76	<0.01	0.02	.96
Ind. bean weight (g)	<0.01	0.01	.93	2.55	0.07	.13	1.64	−0.04	.22	0.04	−0.02	.84
Plant biomass (g)	1.09	−0.61	.31	30.71	2.82	**<.001**	6.42	−0.78	**.020**	0.63	−0.71	.44
Harvest index	1.22	−0.14	.29	0.22	−0.06	.65	0.03	−0.08	.87	3.31	0.17	.084
Root biomass (g)	0.30	−0.08	.59	19.99	0.52	**<.001**	2.07	−0.15	.17	<0.01	−0.02	.93
Root length (cm)	4.03	−1.74	.063	0.30	0.33	.59	0.18	0.25	.68	<0.01	0.10	.94
Aphid category	0.48	0.23	.50	‐	‐	‐	‐	‐	‐	‐	‐	‐
Flower abundance	‐	‐	‐	20.98	6.70	**<.001**	‐	‐	‐	‐	‐	‐
log Poll per flower	‐	‐	‐	0.83	0.27	.38	‐	‐	‐	‐	‐	‐
log Rob per flower	‐	‐	‐	0.14	−0.17	.71	‐	‐	‐	‐	‐	‐

*P*‐ values in bold are significant at the .05 level.

Bean mass per plant was explained by an interaction between water availability and insect herbivory. Aphid herbivory reduced bean mass per plant by 80% in well‐watered plants and water stress reduced yield by 57% in the absence of aphid herbivory. However, the combined effect of aphid herbivory and water stress reduced bean mass per plant less (86%) than the sum of the individual effects (Figure 2d; Table 1). In addition, an interaction between pollination and insect herbivory explained bean mass per plant. Aphid herbivory reduced bean mass per plant by 83% in self‐pollinated plants but insect pollination increased bean mass per plant by 61% in the absence of aphid herbivory. However, the combined effects of aphid herbivory and insect pollination reduced yield more (53%) than the sum of the individual effects (Figure 2e; Table 1). Insect pollination increased bean mass per plant independently of water availability by 78% (Figure 2f; Table 1).

### 3.3 Pods per plant, beans per pod and individual bean weight

The number of pods per plant was also determined by an interaction between water availability and insect herbivory. Aphid herbivory reduced the number of pods by 67% in well‐watered plants and water stress reduced the number of pods per plant by 46% in the absence of aphid herbivory. However, the combined effect of aphid herbivory and water stress reduced the number of pods less (80%) than the sum of the individual effects (Figure 2g; Table 1). Insect pollination increased the number of pods per plant by 42% independently of insect herbivory (Figure 2h) and water availability (Figure 2i; Table 1).

**FIGURE 3:**
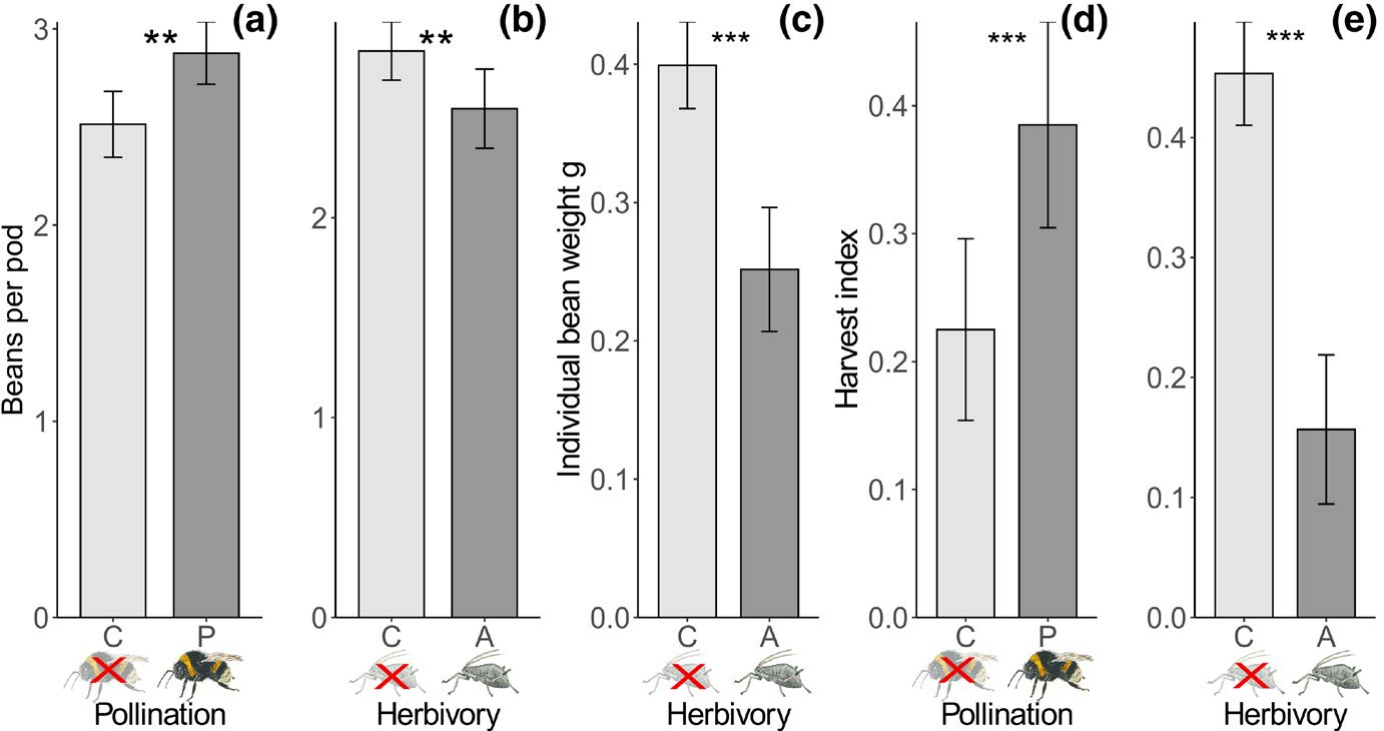
The number of beans per pod (a, b), individual bean weight (grams; c) and harvest index (d, e) in relation to herbivory and pollination. Aphid‐infested (aphid symbol), aphid‐free (crossed‐out aphid symbol). Insect pollination (bumble bee symbol), self‐pollination (crossed‐out bumble bees). Alpha‐levels are indicated by: ***<0.001; **<0.01 and whiskers represent 95% confidence intervals

The number of beans per pod was 15% higher in insect‐pollinated plants (Figure 3a) and 10% lower in aphid‐infested plants (Figure 3b; Table 1). Individual bean weight was 38% lower in aphid‐infested plants (Figure 3c; Table 1).

### 3.4 Aboveground plant biomass and harvest index

Aboveground plant biomass was determined by an interaction between water availability and insect herbivory. Aphid herbivory reduced aboveground plant biomass by 43% in well‐watered plants and water stress reduced aboveground plant biomass by 58% in the absence of aphid herbivory. However, the combined effect of water stress and aphid herbivory reduced aboveground plant biomass less (69%) than the sum of the individual effects (Figure 2j; Table 1). In addition, an interaction between pollination and insect herbivory determined aboveground plant biomass. Aphid herbivory reduced aboveground plant biomass by 45% in self‐pollinated plants and insect pollination reduced aboveground plant biomass by 13% in the absence of aphid herbivory. However, the combined effect of insect pollination and aphid herbivory reduced aboveground plant biomass less (39%) than the sum of the individual effects (Figure 2k; Table 1). There was no interaction between pollination and water availability for aboveground plant biomass (Figure 2l; Table 1).

The harvest index was 70% higher in insect‐pollinated plants (Figure 3d; Table 1) and 64% lower in aphid‐infested plants (Figure 3e; Table 1).

### 3.5 Root biomass and root length

Root biomass was determined by an interaction between water availability and insect herbivory. Aphid herbivory reduced root biomass in well‐watered plants by 44% and water stress reduced root biomass by 59% in the absence of aphid herbivory. However, the combined effect water stress and aphid herbivory reduced root biomass less (67%) than the sum of the individual effects (Figure 2m; Table 1). Pollination did not affect root biomass and there were no interactions between pollination and insect herbivory (Figure 2n) or pollination and water availability for root biomass (Figure 2o; Table 1). The decrease in root biomass was mostly driven by a decrease of lateral roots (Figure S4). Root length was not affected by any of the treatments or their interactions (Table 1).

### 3.6 Aphid abundances

Aphid abundances on the inoculated plants steadily increased in both well‐watered and water‐stressed plants, but aphid colonies grew faster on well‐watered plants (Figure S3a). Once the cages were removed 30 days after first inoculation, aphid colonies rapidly declined in abundance, likely due to predation (Figure S3a). Overall, aphid abundances were 16% higher on well‐watered plants compared with water‐stressed plants (Figure S3b; Table 1).

### 3.7 Flower abundance and pollinator visitation rate

**FIGURE 4:**
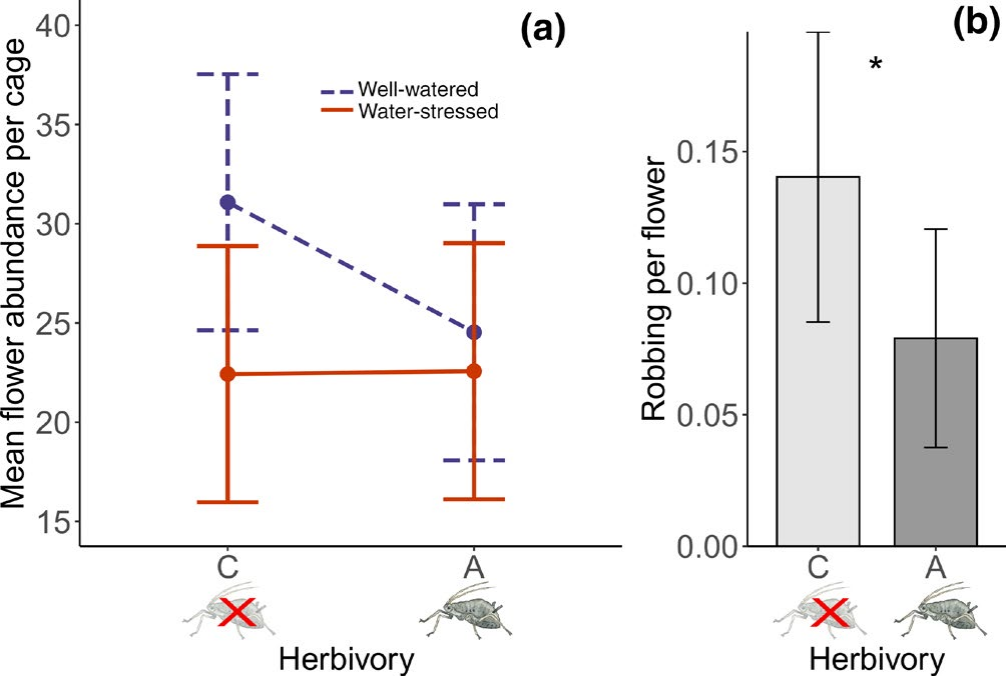
Mean flower abundance per cage in well‐watered plants (blue, dashed line) and water‐stressed plants (orange, continuous line), aphid‐infested (aphid symbol) and aphid‐free (crossed‐out aphid symbol) plants (a). Mean number robbing events per flower over 10 min in aphid‐infested (aphid symbol) and aphid‐free (crossed‐out aphid symbol) plants (b). Alpha‐levels are indicated by: *<0.05 in (b) and whiskers represent 95% confidence intervals

Flower abundance was explained by an interaction between water availability and insect herbivory. Aphid herbivory reduced flower abundance by 21% in well‐watered plants and water stress reduced flower abundance by 28% in the absence of aphid herbivory. However, the combined effect of water stress and aphid herbivory reduced flower abundance less (27%) than the sum of the individual effects (Figure 4a; Table 1). There was no effect of any treatment on the number of legitimate pollination visits per flower (Table 1), but flowers on aphid‐free plants were robbed 77% more often by bumble bees than aphid‐infested plants (Figure 4b; Table 1).

## 4 DISCUSSION

Gradually increasing soil water depletion inducing water stress in combination with insect herbivory interactively shaped faba bean yield. Contrary to our hypothesis, the negative effect of aphid herbivory on flower and pod production, and ultimately bean mass per plant and yield per hectare, was more pronounced in well‐watered plants. The absolute benefit of insect pollination on yield and its components was largely independent of water availability and insect herbivory. The independent benefit of insect pollination on yield also contrasts our hypothesis, where we expected stressed plants to benefit less from insect pollination due to changes in quality and quantity of floral rewards that lead to reduced pollinator visitation rate and changed foraging behaviours.

### 4.1 Interactions between abiotic and biotic climate change stressors

The interaction between water availability and insect herbivory on yield components, flower abundance, aboveground plant biomass and root biomass was likely driven by aphid abundances reaching higher numbers on well‐watered plants. Our finding that aphid abundances are higher in well‐watered compared with water‐stressed plants, contradicts the original plant stress hypothesis, which states that plant host stress enhances insect herbivore population growth (White, 1969). Empirical experiments have, however, shown that aphid population growth can be higher on water‐stressed (Fereres et al., 1988; Oswald & Brewer, 1997), well‐watered (Mody et al., 2009; Oswald & Brewer, 1997; Simpson et al., 2012) and intermediately water‐stressed plants (Tariq et al., 2012) depending on aphid species, host plant and water stress regime. The increased aphid population growth we observed was likely due to aphids benefitting from feeding on well‐watered, vigorously growing plants with higher quality phloem composition (Hale et al., 2003; Mcvean & Dixon, 2001; Mody et al., 2009). In contrast, aphids on water‐stressed plants might have suffered from reduced feeding efficiency due to low plant turgor pressure and more viscous sap (Huberty & Denno, 2004; Kennedy et al., 1958). The fact that the interaction between water availability and insect herbivory was already present for flower abundance and was then maintained to crop yield, illustrates that the interaction between water availability and insect herbivory for plant reproduction was established at an early plant growth stage. Plants subjected to aphid herbivory increase transpiration rates as their stomata open up causing increased water loss (Shannag, 2007). Thus, in our experiment, increased aphid herbivory on well‐watered plants potentially hastened soil water uptake, and led to lower resource allocation into flower production already at an early growth stage.

### 4.2 The pollination benefit following plant biotic and abiotic stress

Insect pollination increased yield, the number of pods per plant and the number of beans per pod independently of herbivory and water availability. The additive benefit of insect pollination is in line with our finding that the rate of legitimate pollinator flower visits was independent of water availability and herbivory. However, while on an absolute scale, the benefit of insect pollination on yield was constant irrespective of whether plants were water‐stressed or well‐watered, the relative benefit of insect pollination was greater in water‐stressed (53%) compared to well‐watered plants (37%). Higher proportional benefits of insect pollination on yield components have been found in intermediately heat‐stressed (Bishop et al., 2016) and water‐stressed (Stoddard, 1986) faba bean plants.

Bean mass per plant was higher in insect‐pollinated compared with self‐pollinated plants, and this insect pollination benefit was higher for aphid‐free plants. Similar interactions between insect pollination and insect herbivory have been found for several crops (Grass et al., 2018; Lundin et al., 2013; Sutter & Albrecht, 2016; van Gils et al., 2016). Possible mechanisms are that herbivore‐infested plants are less attractive to pollinators due to a reduced ability to invest into floral rewards, or, alternatively, increased defence mechanisms reducing the quality or palatability of floral rewards (Jacobsen & Raguso, 2018). However, we found no evidence that bumble bees legitimately visited aphid‐infested plants less frequently, suggesting that the synergistic interaction was not driven by reduced insect pollination. Nevertheless, bumble bees more often robbed flowers on aphid‐free plants. While nectar robbing does not contribute to pollen outcrossing, it can enhance self fertilization by tripping the faba bean flowers (Kendall & Smith, 1975; Soper, 1952). It is thus possible that the synergistic interaction determining bean mass per plant was driven by a nectar‐robbing behaviour that enhanced self‐pollination. The increased nectar robbing on aphid‐free plants could be caused by aphid herbivory reducing the quantity and quality of the nectar, but not of the pollen, making aphid‐infested plants less attractive for pollinators to rob nectar from. Alternatively, it is possible that aphid‐free plants had more resources available to invest such that more fertilized ovules, as a consequence of insect pollination, developed into mature seeds (Lee, 1988).

Aboveground biomass was affected by a negative interaction between insect herbivory and pollination treatments, where the negative effect of aphid herbivory on aboveground plant biomass was reduced in insect‐pollinated plants, which was also reflected in the harvest index. This negative interaction was likely due to insect‐pollinated plants allocating resources into seed formation and shifting away from allocating resources into plant growth, while plants not visited by pollinators continued to grow and produce flowers (Adamidis et al., 2019), in particular when not affected by herbivory.

### 4.3 Plant responses to multiple stressors and ecosystem services under climate change

In order to survive and reproduce, flowering plants need to respond to biotic and abiotic cues by constantly fine‐tuning their resource allocations to balance the conflicting selective pressures of herbivore defences, water stress avoidance and pollinator attraction (Jacobsen & Raguso, 2018; Suzuki et al., 2014; Zhang & Sonnewald, 2017). Gaining a better understanding of how abiotic and biotic stressors interact with each other and modulate the benefits of ecosystem services such as pollination, is essential to maximize crop production under current and future climatic conditions. We simulated a long period without precipitation and ensuing plant water stress in line with the region's climate change predictions. Simulated heat waves were shown to modulate the insect pollination dependence of potted faba bean plants (Bishop et al., 2016, 2017). However, there are many other climate change scenarios that induce abiotic stress to plants, such as the combination of extended periods of no precipitation and elevated temperature or more complex patterns stemming from precipitation intensity and frequency that would need to be explored under realistic field conditions for their impacts on yield. In addition, we subjected the crop to one aphid species, but plants respond differently to different aphid species (Oswald & Brewer, 1997; Tariq et al., 2012). Furthermore, aphids are phloem‐sucking herbivores, but plant responses can differ for other types of insect herbivory, such as leaf‐chewing, xylem feeding and stem boring (Mody et al., 2009). Interactions between different types of abiotic and biotic stressors might thus result in very different crop yield outcomes.

## 5 CONCLUSION

Crop production is subject to a range of abiotic and biotic stressors that interact with ecosystem services, all of which are influenced by climate change. In our case, water stress and aphid herbivory interactively shaped faba bean yield. Individually, aphid herbivory reduced yield by 79% and water stress reduced yield by 52%. However, the combined effect of water stress and aphid herbivory reduced yield less (84%) than the sum of the individual stressor effects. Our results suggest that large yield gains can only be achieved when both water stress and aphid herbivory are mitigated simultaneously. This interactive effect has important implications for crop management under climate change. Water resources are limited and freshwater withdrawal for irrigation needs to be minimized. Hence, crop yield benefits will be maximized if use of irrigation is prioritized in crops with high levels of herbivore control. Similarly, because the yield loss caused by aphid herbivory was lower under water stress, the threshold for pesticide use against insect herbivores will be higher under water stress. Insect pollination had a constant positive effect on faba bean yield (+68%), irrespective of water stress and insect herbivory, highlighting the importance of managing agricultural landscapes to support pollinator communities irrespective of water stress and insect herbivory pressure. Evaluating the potential of insect pollination to increase yields under climate change is particularly pertinent considering that pollinator communities themselves are threatened by climate change (Soroye et al., 2020). In order to ensure sustainable agriculture under climate change, where the use of agronomic inputs such as irrigation water and pesticides are minimized and reserved to cases where their effects on crop yield are critical, it is essential to gain a better understanding of how abiotic and biotic stressors interact with each other and with ecosystem services such as pollination.

## Supporting information

Supplementary Material

## Data Availability

The data that support the findings of this study are openly available in Dryad at https://doi.org/10.5061/dryad.gqnk98skd.
