# Supplementary material for: Water stress and insect herbivory interactively reduce crop yield while the insect pollination benefit is conserved
Source: Glob Chang Biol. 2020 Oct 29;27(1):71–83. doi: 10.1111/gcb.15386 (PMC7756552; doi:10.1111/gcb.15386)
Supplement: Supplementary file 1 — Supplementary Material [file GCB-27-71-s001.docx]

**Table S1** Summary of local climatic conditions: long-term averages (1950-2019) and conditions relative to 2019, including the well-watered and water stressed treatments. Meteorological data were extracted from the EObs gridded dataset at 0.01 degree spatial resolution, version 21.0e (Cornes, van der Schrier, van den Besselaar, & Jones, 2018). In parenthesis is the amount of water we irrigated.

|  | Temperature (°C) | | Total water inputs (and irrigation amount, if any) (mm) | | |
| --- | --- | --- | --- | --- | --- |
|  | Long-term mean | 2019 | Long term mean precipitation | well-watered treatment | water-stressed treatment |
| May | 10.3 | 10.3 | 33.9 | 57.0 (0) | 57.0 (0) |
| June | 14.8 | 17.5 | 51.1 | 32.0 (32.0) | 0 (0) |
| July | 17.0 | 16.4 | 66.9 | 66.5 (52.5) | 14.0 (0) |
| August | 15.8 | 17.0 | 69.9 | 68.0 (0) | 68.0 (0) |
| May-August | 14.5 | 15.3 | 221.8 | 223.5 (84.5) | 139.0 (0) |

| 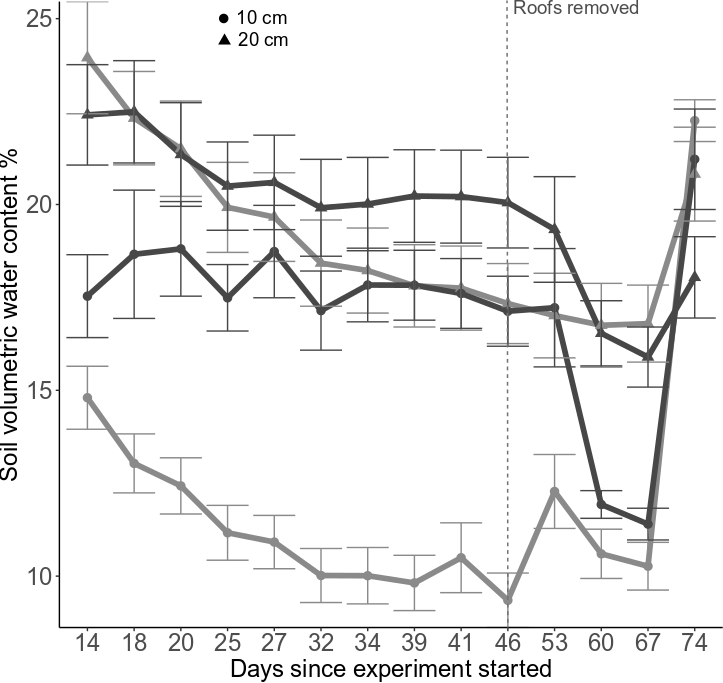 |
| --- |
| **Figure S1** Soil volumetric water content (%) against days since the experiment started for well-watered treatments (light grey) and water-stressed treatments (dark grey) at 10 cm (circles) and 20 cm (triangles) soil depths. Vertical dashed line indicated when the roofs were removed and both treatments became rainfed again. Whiskers represent standard errors. |
|  |
|  |
|  |

| 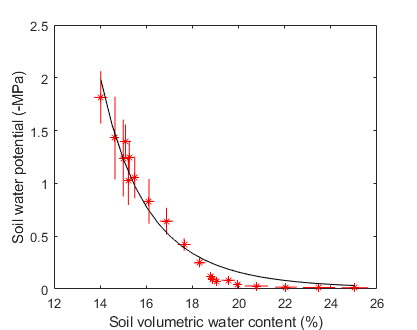 |
| --- |
| **Figure S2** Soil water retention curve based on co-located measurements of soil water potential (y axis) and soil volumetric water content (x axis), relative to 20 days. Symbols represent daily averages of 30 min measurements; whiskers extend over ± one standard deviation. We removed the first part of the record after installation (10 days), i.e., the time preceding the first rainfall event (when soil contact was likely poor) and the initial part of the ensuing dry down (when soil water potentials were too close to zero for the MPS-6 ceramic block to return accurate readings). The black line is the reconstructed water retention curve $\psi=\psi_{sat}\theta^{-b}$ (Clapp & Hornberger, 1978) based on the fitting of the daily averages ($\psi_{sat}$=-1.7 10^-6^ MPa, $b$=7.1). Based on that, the volumetric water content at -1.5 MPa (i.e., at wilting point) corresponds to 14.6%. |


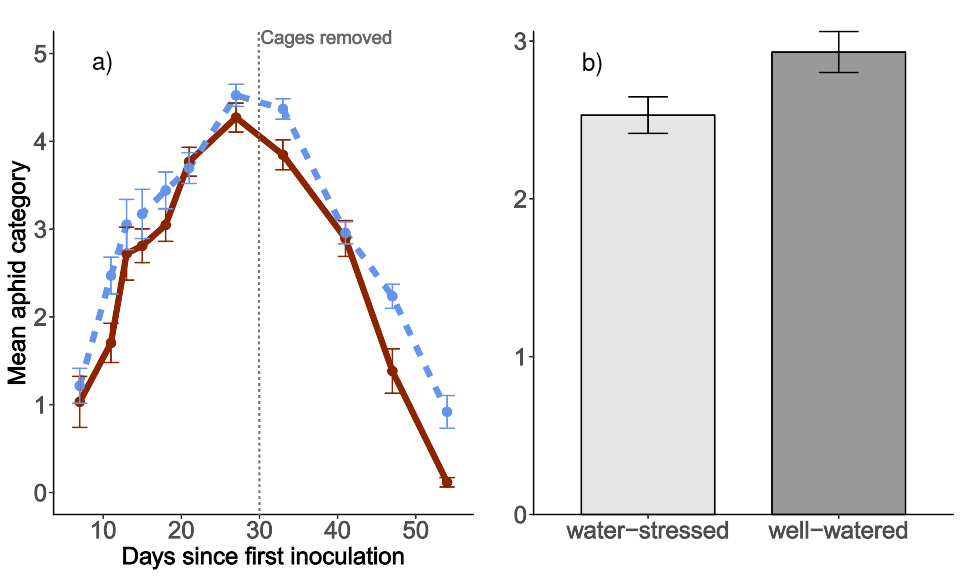


**Figure S3** Mean aphid infestation categories in the well-watered (blue, dashed) and water-stressed (brown, solid) treatment at each sampling round a). Mean aphid infestation categories for water-stressed and well-watered treatment across all sampling occasions b). Dashed black line in a) indicates when cages were removed and aphids were subjected to biocontrol by natural enemies. Whiskers represent standard errors.

| 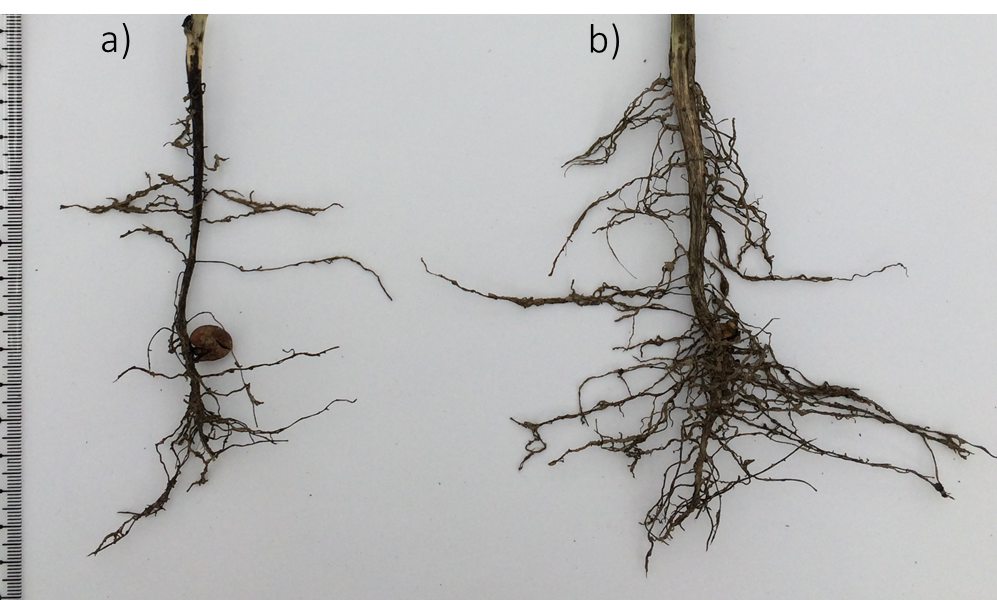 |
| --- |
| **Figure S4** Photograph of root system of a water-stressed a) and well-watered faba bean plant b). Scale in mm. |
